# Supplementary figures and images for: Resting mitochondrial complex I from Drosophila melanogaster adopts a helix-locked state
Source: eLife. 2023 Mar 23;12:e84415. doi: 10.7554/eLife.84415 (PMC10036122; doi:10.7554/eLife.84415)

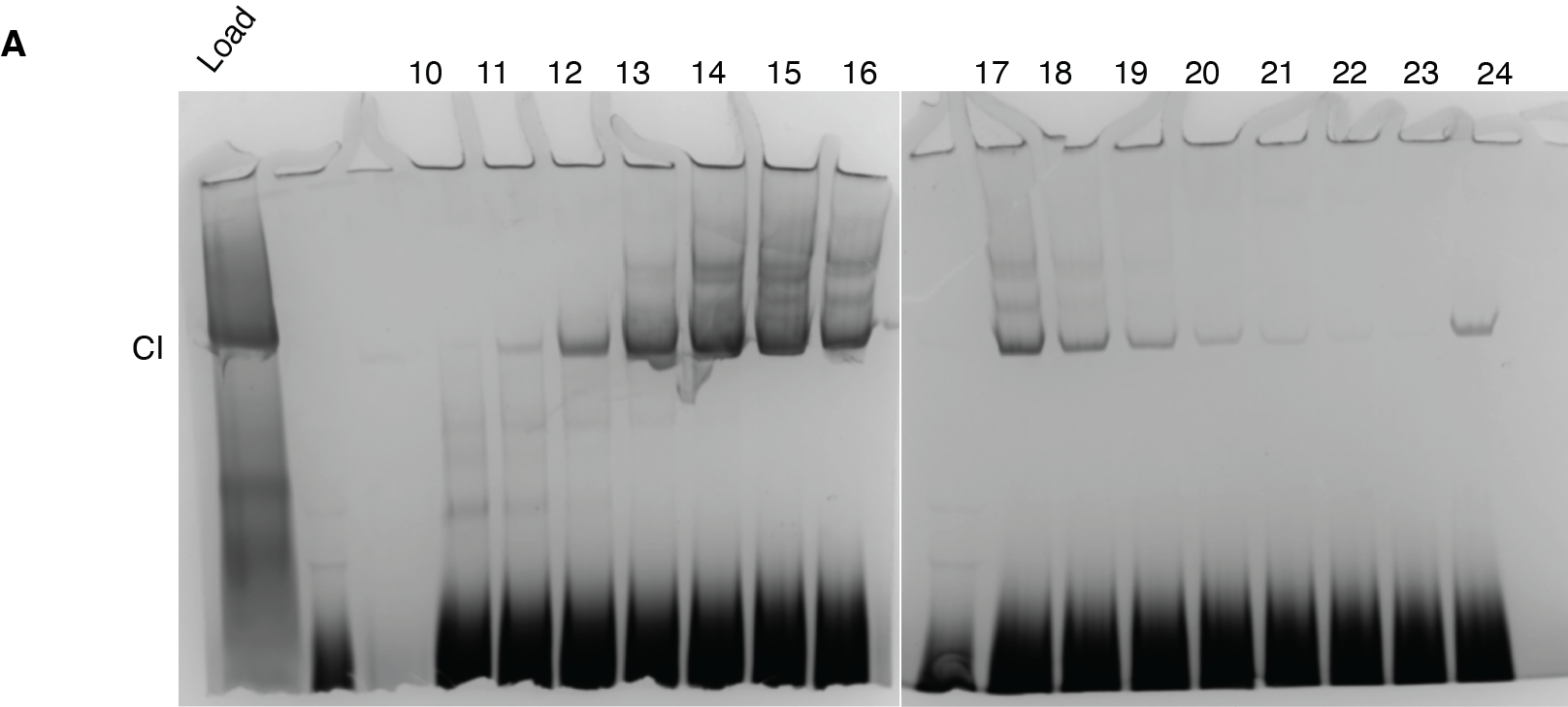

Supplement: Figure 1—figure supplement 1—source data 1. [file elife-84415-fig1-figsupp1-data1.zip › Figure 1-figure supplement-source data 1/Figure 1-figure supplement 1-source data 1.png]

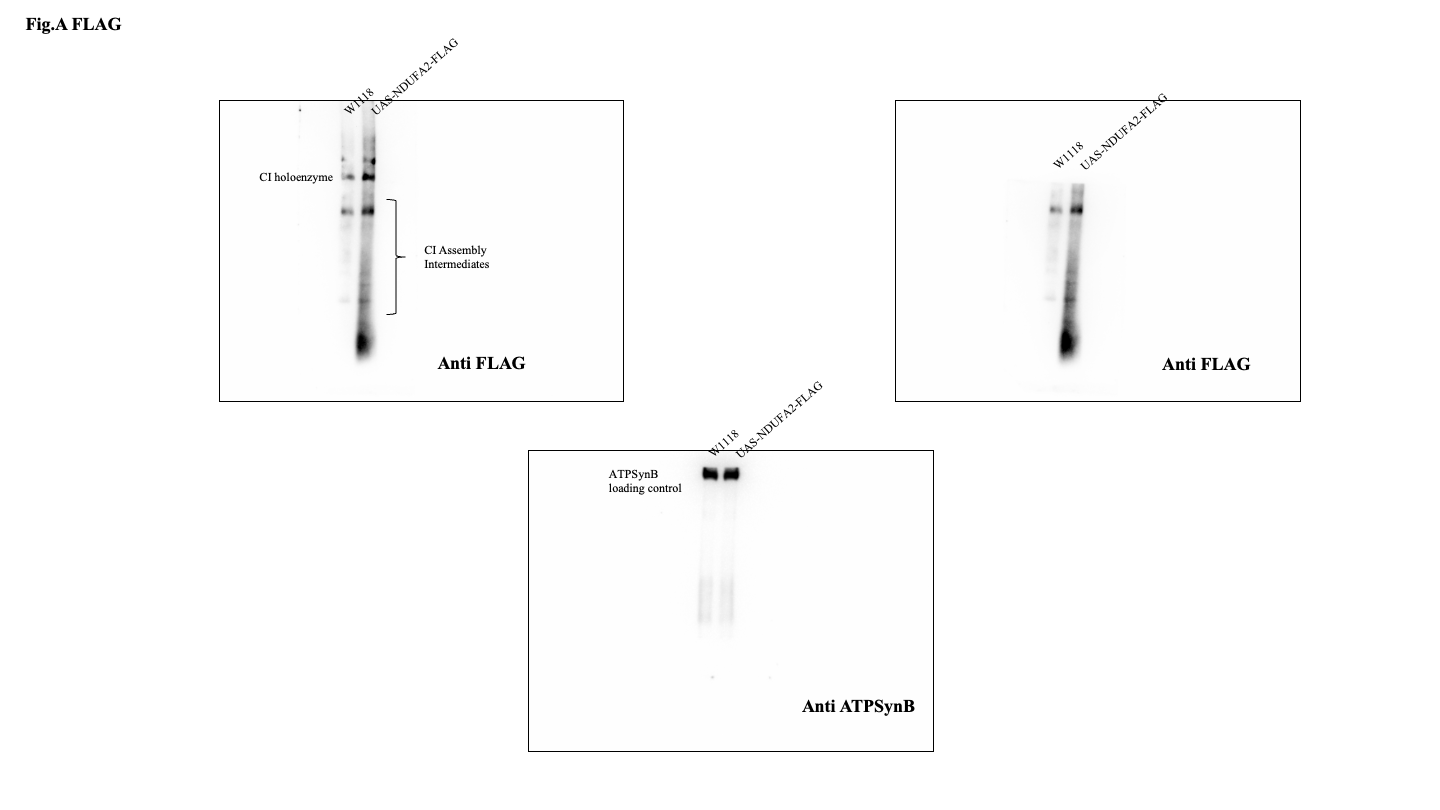

Supplement: Figure 1—figure supplement 5—source data 1. — Panels A and B are biological replicates. [file elife-84415-fig1-figsupp5-data1.zip › Figure 1-figure supplement 5-source data 1/Slide3.png]

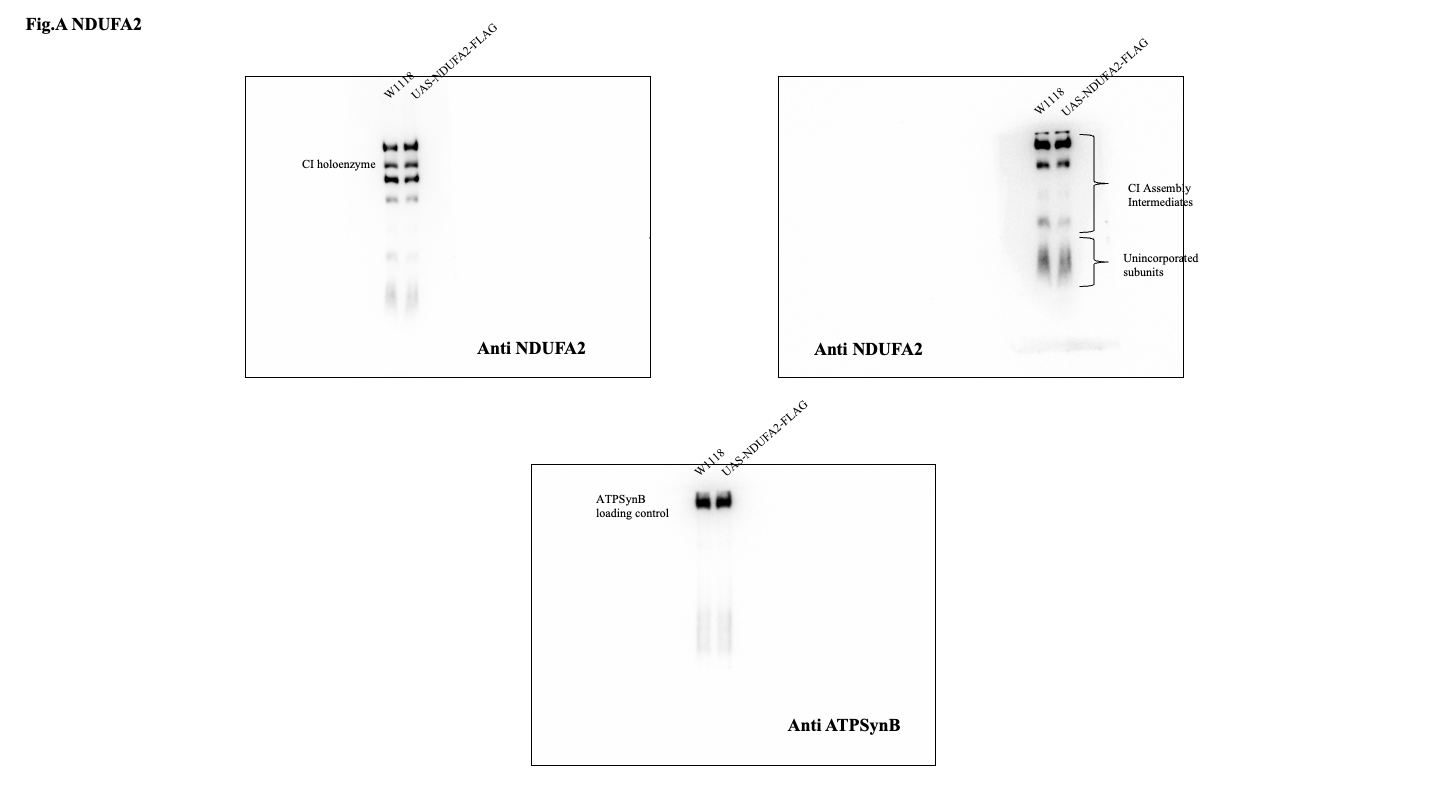

Supplement: Figure 1—figure supplement 5—source data 1. — Panels A and B are biological replicates. [file elife-84415-fig1-figsupp5-data1.zip › Figure 1-figure supplement 5-source data 1/Slide2.png]

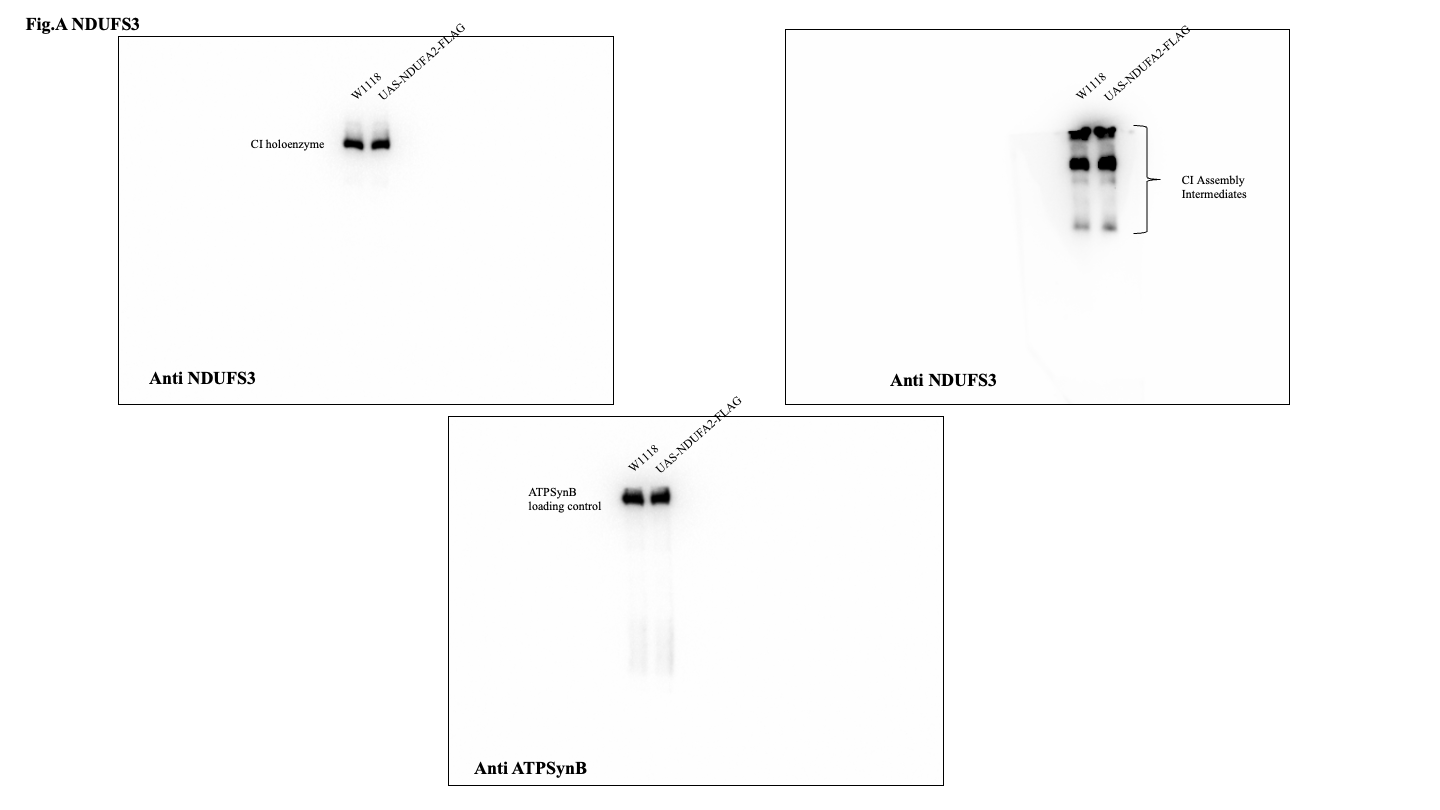

Supplement: Figure 1—figure supplement 5—source data 1. — Panels A and B are biological replicates. [file elife-84415-fig1-figsupp5-data1.zip › Figure 1-figure supplement 5-source data 1/Slide1.png]

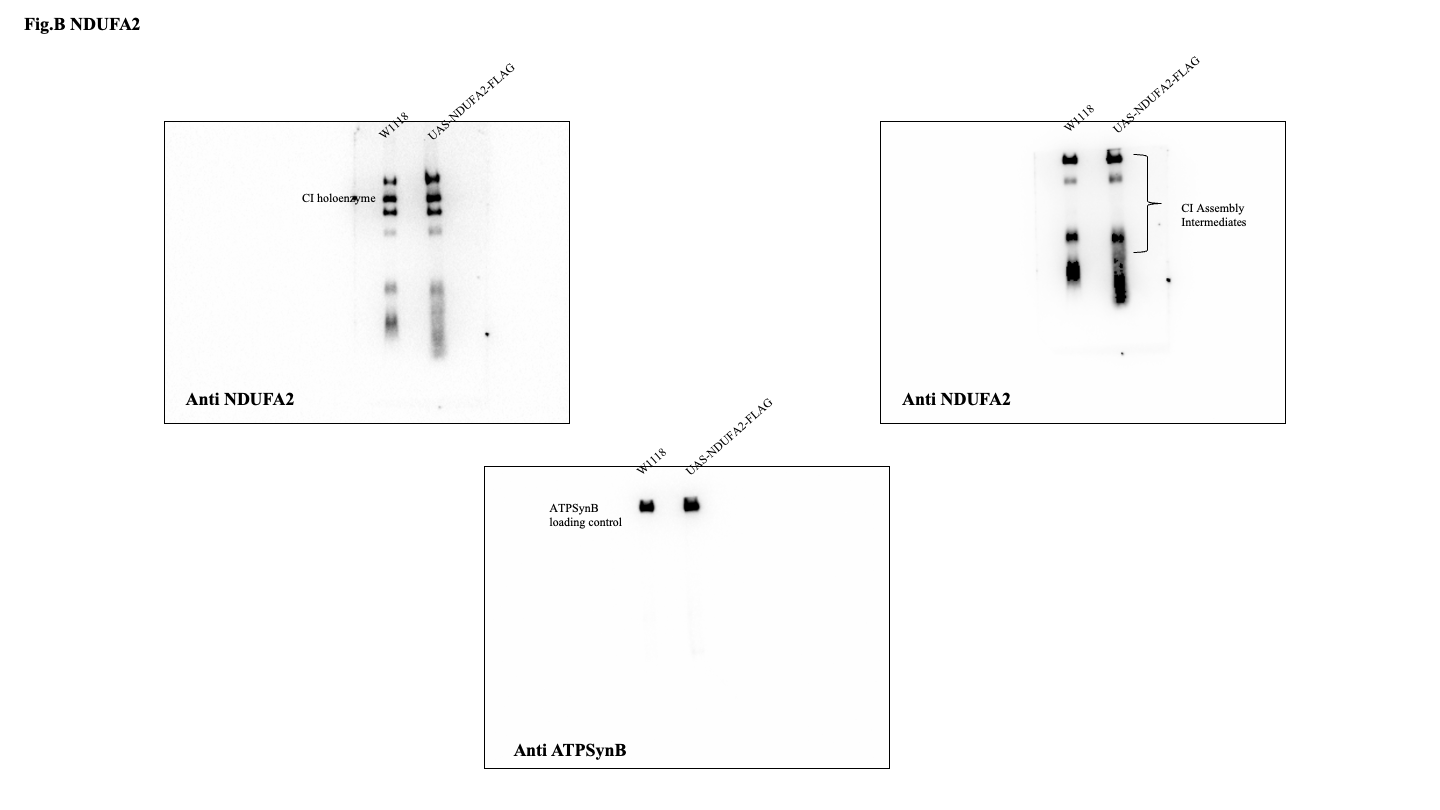

Supplement: Figure 1—figure supplement 5—source data 1. — Panels A and B are biological replicates. [file elife-84415-fig1-figsupp5-data1.zip › Figure 1-figure supplement 5-source data 1/Slide5.png]

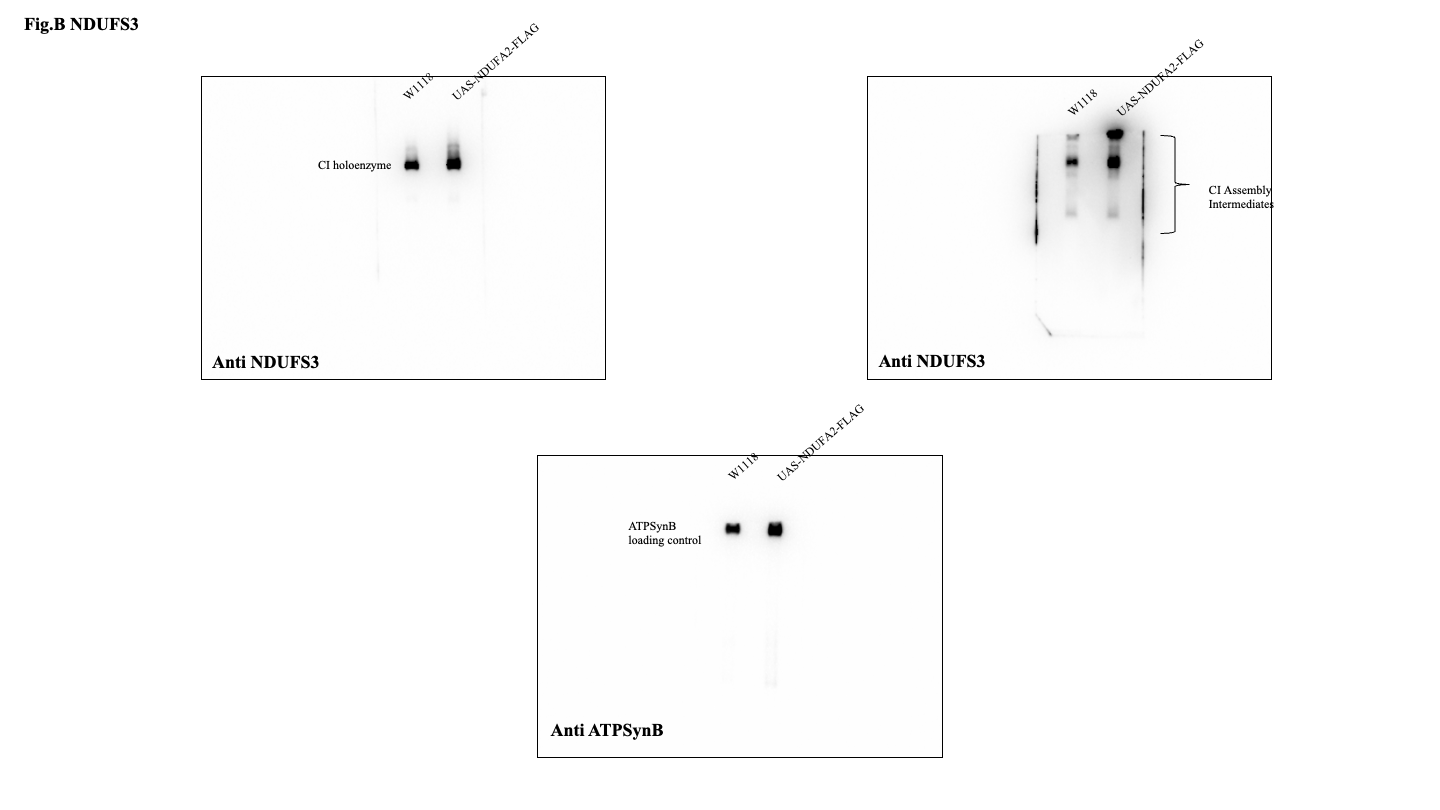

Supplement: Figure 1—figure supplement 5—source data 1. — Panels A and B are biological replicates. [file elife-84415-fig1-figsupp5-data1.zip › Figure 1-figure supplement 5-source data 1/Slide4.png]

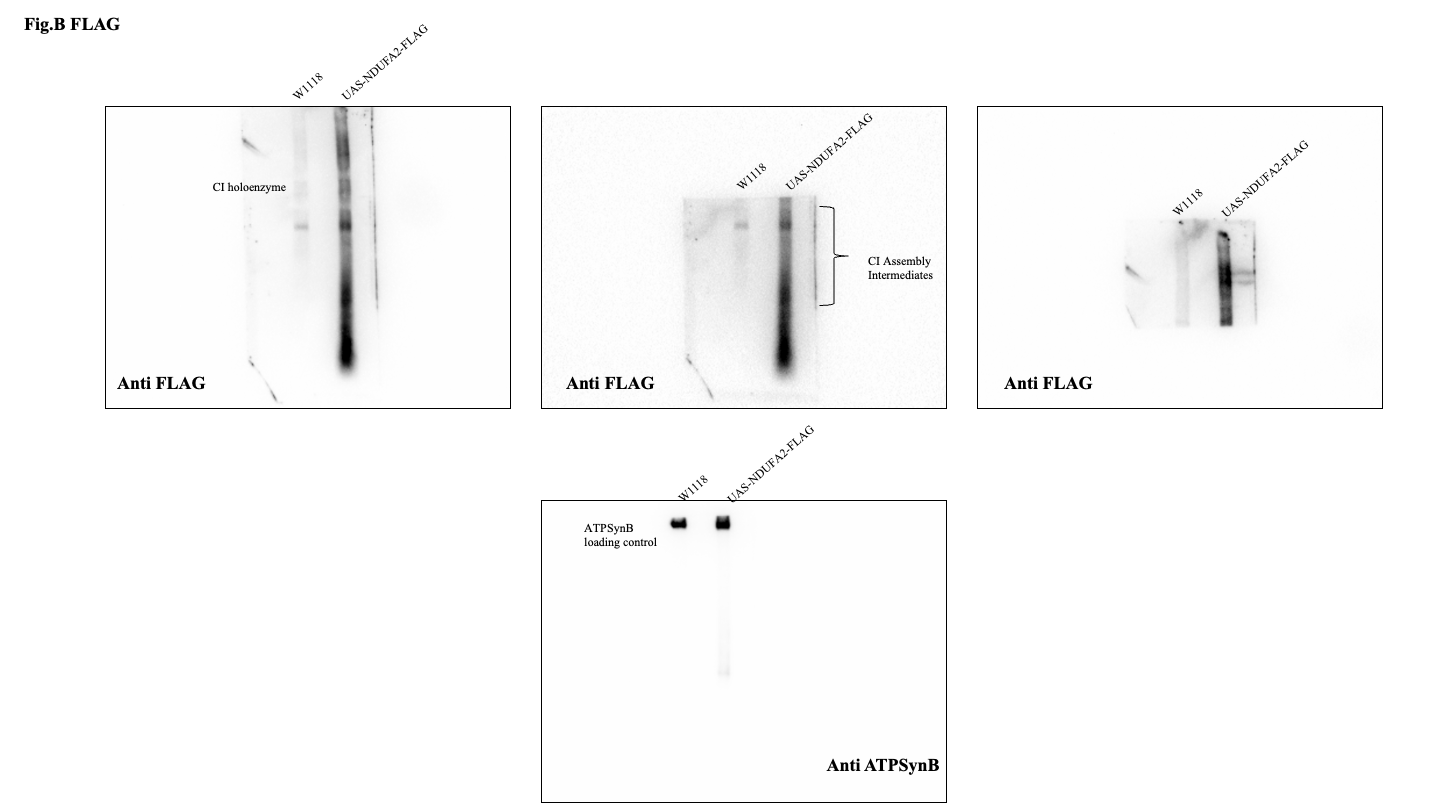

Supplement: Figure 1—figure supplement 5—source data 1. — Panels A and B are biological replicates. [file elife-84415-fig1-figsupp5-data1.zip › Figure 1-figure supplement 5-source data 1/Slide6.png]
